# 5-HTTLPR polymorphism and anxious preoccupation in early breast cancer patients

**DOI:** 10.2478/v10019-012-0024-0

**Published:** 2012-11-09

**Authors:** Giulia Schillani, Daniel Era, Tania Cristante, Giorgio Mustacchi, Martina Richiardi, Luigi Grassi, Tullio Giraldi

**Affiliations:** 1 Department of Life Sciences, University of Trieste, Trieste, Italy; 2 University of Trieste and Centro Sociale Oncologico, ASS1, Trieste, Italy; 3 Section of Psychiatry, Department of Behavior and Communication, University of Ferrara, Ferrara, Italy

**Keywords:** breast cancer, mental adjustment to cancer, depression, anxiety, 5-HTTLPR polymorphism

## Abstract

**Background:**

Difficulties in coping with cancer, and the accompanying anxious and depressive symptoms, have been shown to affect the mood and the quality of life in breast cancer patients. 5-Hydroxytryptamine Transporter Gene-linked Polymorphic Region (5-HTTLPR) functional polymorphism of serotonin transporter has been shown to influence the adaptation to stressful life events. The aim of this prospective study was therefore to examine the association of 5-HTTLPR with the mental adaptation to cancer diagnosis and treatment.

**Patients and methods:**

Forty eight consecutive patients with early mammary carcinoma were evaluated at enrolment and at follow up after one and three months. The patients were characterized psychometrically using the Hospital Anxiety and Depression Scale (HADS) and the Mini-Mental Adjustment to Cancer Scale (Mini-MAC); 5-HTTLPR allelic variants were determined using PCR-based techniques.

**Results:**

In women with early breast cancer, the mental adaptation to the disease was associated with high scores of avoidance and anxious preoccupation of Mini-MAC, which decreased with time at follow up. Anxious preoccupation decreased with time less in patients with the S/S and S/L genetic variant of 5-HTTLPR as compared with the L/L carriers (*p*=0.023), indicating gene - environment interactions.

**Conclusions:**

These results indicate that the characterization of 5-HTTLPR allows the identification of breast cancer patients in greater risk of mental suffering, for which specific intervention may be focused; in case of drug therapy, they provide indications for the choice of most appropriate agent in a pharmacogenetic perspective.

## Introduction

A large literature indicates that facing a neoplastic disease is a challenging experience which deeply involves cognitive and emotional aspects of the individual.^1^–^12^ Coping with cancer requires a mental adaptation to the communication of diagnosis, to the choice between the alternatives for the subsequent adjuvant treatment, and to the follow-up. Specific modalities of mental adaptation to cancer, namely hopelessness-helplessness, fighting spirit, fatalism, avoidance and anxious preoccupation, have been shown by Greer *et al.* and Watson *et al.* to characterize the individual modalities of coping with the disease.^13^,^14^ While these modalities have evident implications for the quality of life of the patients, these investigators also reported an increased risk of death in women with high scores on the hopelessness-helplessness category of the Mental Adjustment to Cancer (MAC) Scale, and also on the Hospital Anxiety and Depression Scale (HADS) category of depression.^13^,^14^

The role of the genetic polymorphism in the response to treatment and survival for oncological patients is well known.^15^,^16^ However, the genetic polymorphism of serotonin transporter (SERT) in causing an increased risk of depression in subjects who experienced stressful life events is also known and it has been initially reported by Caspi *et al..*^17^ In the prospective-longitudinal study of a representative birth cohort, the authors tested why stressful experiences lead to depression only in some subjects. A functional polymorphism, consisting in one or two copies of the short (S) allele of the 5-HTTLPR (5-Hydroxytryptamine Transporter Gene-linked Polymorphic Region) in the promoter region of the SERT gene, was found to increase the influence of stressful life events on the development of depression, representing gene - environment interactions.^17^ A large number of investigations have subsequently examined the associations of the development of mental disorders in a variety of psychological and psychiatric conditions with SERT polymorphism, evaluating the role played by the allelic variants of 5-HTTLPR, which are endowed with high (L/L) or low (S/S, S/L) functional activity and have a high penetrance in the populations examined.^18^

The most frequent oncological disease in women is breast cancer^19^,^20^ and the communication of breast cancer diagnosis, as well as the treatments subsequently performed, has been shown to be a constellation of significant stressful events, requiring adequate psychological adaptation by patients.^1^–^5^ Different modalities of mental adaptation to cancer have been identified^6^, and may affect the quality of life^7^–^12^ and even the prognosis of the disease.^21^ The mental adjustment to cancer has been extensively investigated by Greer *et al.* in terms of the coping strategies adopted by patients.^13^ An initial prospective study of subjects with early breast cancer showed that the disease free survival at five years was significantly more frequent among those women who initially reacted by denial or fighting spirit, rather than by stoic acceptance or feelings of helplessness and hopelessness.^13^ In a larger cohort subsequently examined, helplessness-hopelessness identified using the MAC scale, and depression measured by the HADS scale, were shown to be significant prognostic factors for the decreased survival.^14^

The aim of the present prospective study has been therefore to examine in women with early breast cancer the role of 5-HTTLPR polymorphism in relation to mental adaptation to cancer, and to depression and anxiety. A group of women who had received the diagnosis of mammary carcinoma, and had been treated with surgery and adjuvant therapy, has been genotypically characterized for 5-HTTLPR. The presence of the functional A〉G variation within the L allele of SERT polymorphism, which has been reported to modulate the role of 5-HTTLPR for depression^22^,^23^, has been also examined. At the time of recruitment and at follow-up one and three months later, these patients were psychometrically characterized using Mini-MAC for evaluating their mental adaptation to cancer, and HADS for assessing depression and anxiety. The data obtained have been analyzed in order to identify the occurrence of associations between the psychometric variables obtained and the patients’ 5-HTTLPR genotypic characteristics of the patients; the results obtained are hereafter reported.

## Patients and methods

The study population consisted of women who received a diagnosis of mammary carcinoma, and who were referred to the Centro Sociale Oncologico, Azienda Servizi Sanitari 1, Trieste, Italy, between February 2008 and August 2009. The patients were recruited after the communication of the cancer diagnosis and surgery and before the beginning of adjuvant treatment (average time 135±9.7 days), and were evaluated at enrolment into the study (T0), and at follow up one (T1) and three (T2) months later.

The study was carried out in accordance with the Declaration of Helsinki and Good Clinical Practice Guidelines, after having been approved by the relevant institutional Ethical Committee, and having received the informed consent by each participant. Conflicts of interest do not appear to exist according to the statements of the authors.

The subjects were characterized psychometrically by a trained psychologist using the Hospital Anxiety and Depression Scale (HADS)^24^, and the Mini Mental Adjustment to Cancer Scale (Mini-MAC)^10^ to examine the mechanisms of psychological adaptation to the disease. HADS is a 14-item questionnaire measuring anxiety (7 items) and depression (7 items), and is designed for the use in medical outpatients. HADS has been used in general psychiatry^25^ and in cancer settings indicating its usefulness, reliability and validity. The Mini-MAC is a 29-item questionnaire measuring patients’ coping with cancer (fighting spirit, avoidance, hopelessness, anxious preoccupation and fatalism) The Mini-MAC was chosen as the primary outcome measure and the validated Italian version of mini-MAC was employed in this study.^10^,^11^

The patients were also characterized for the 5-HTTLPR polymorphism as described below. Subjects older than 75 years, and those with a previous history of psychopathology such as psychotic or endogenous major depressive disorder, were not included in the study. For each patient, the demographic, as well as the previous and current medical history, were recorded for the later analysis.

Genomic DNA was obtained from whole blood or buccal cells, using standard procedures (MasterAmp^TM^ buccal swab brushes, Epicentre Technologies; GenElute^TM^ blood Genomic DNA Kit, Sigma).

Polymerase chain reaction (PCR) amplification of the DNA region of 5-HTTLPR was performed using the primers described by Gelernter *et al.*^26^, and with the GC-Rich PCR System (Roche Molecular Biomedicals) in a 50-μL reaction containing 20–100 ng of DNA; 100 μm deoxyribonucleoside triphosphate (dNTPs), 20 pmol for each primer, and 1.5 mM MgCl_2_. DNA was denatured at 95°C for 10 min and subjected to 40 cycles of 40s denaturation at 94°C, 45s annealing at 56°C, 40s extension at 72°C, and 10 min final extension at 72°C. The products of PCR amplification were separated on a 2% agarose gel, and were visualised in ultraviolet light after ethidium bromide staining.

The presence of the functional A〉G variation in the long (L) allele identified by Hu *et al.*^23^ as the La and Lg alleles, was also evaluated by digestion of the PCR products with the Hpa II enzyme. The digested mixture was size-fractioned on agarose gel and visualised in ultraviolet light after ethidium bromide staining.

A statistical analysis was carried out using descriptive statistic, Pearson correlation and Analysis of Variance (ANOVA) to characterize the sample and to evaluate the relationships of the psychometric scales scores as a function of follow-up time and of 5-HTTLPR genotypic variants, using the SPSS 13.0 package (SPSS Inc. Chicago, IL, USA), as indicated in the Tables. Statistical significance was set at the *p*< 0.05 level.

## Results

The patients’ socio-demographic data and clinical characteristics are illustrated in Table 1. The patients considered were initially subjected at recruitment to psychometrical evaluation (T0: N=48), and were re-tested psychometrically at follow up one month after recruitment (T1: N=48); a third evaluation (T2: N=35) three months after T0 could be performed on 35 (72.9%) of the initial 48 patients.

The allelic variants of 5-HTTLPR polymorphism of the subjects considered are shown in Table 2. None of the patients had an Lg allele; the genotypic distribution for 5-HTTLPR did not significantly differ from the Hardy–Weinberg equilibrium (*χ*^2^=2.30; *p*=0.13).

The psychometric measures performed were initially analyzed to determine the possible existence of difference attributable to the socio-demographic and clinical characteristics of the patients. When the scores of subscale employed were stratified for age, employment and marital status, education, disease stage and treatment, no significant differences were observed at recruitment (T0). Six out of 48 patients were receiving at recruitment an antidepressant treatment which had been prescribed before recruitment into this study, and 12 patients were treated with benzodiazepines. These groups displayed at T0 and T1 scores for depression (HADS) higher than the untreated ones; in both cases, the difference was not significant also when examined at follow up. Any reduction with time of anxiety and depression observed during the present study cannot consequently be ascribed to differences existing at recruitment, and to the drug treatments performed.

The data obtained using Mini-MAC are reported in Table 3. The scores of Mini-MAC sub-scale for anxious preoccupation displayed a decrease with time of follow up, and the analysis with ANOVA showed a significant effect of time (*F*=5.646, *df*=2.128, *p*=0.004) and genotype (*F*=5.296, *df*=2.128, *p*=0.023). For Mini-MAC avoidance scores, ANOVA showed a significant effect of time only (*F*=3.107, *df*=2.128, *p*=0.048).

The psychometric scores obtained using HADS at T0, T1 and T2 are illustrated in Table 4. For both HADS subscales, no statistically significant effects of time and genotype were observed.

## Discussion

The initial findings of Caspi *et al.* on the role played by the 5-HTTLPR genetic polymorphism of serotonin transporter in modulating the appearance of depression after stressful life-events was initially not supported by the results of the meta-analysis performed in 2009 by Riesch *et al*.^27^, and by Munafò *et al*..^28^ Moreover, Middeldorp *et al*., in a study conducted on series of twins, were unable to demonstrate any significant interaction between 5-HTTLPR polymorphism and the number of life events experienced across the life span or the year preceding the depressive episode.^29^ A subsequent meta-analysis, where the small number of studies previously considered was increased has been recently published, provided support, in contrast to the results of the these earlier studies, for the hypothesis that 5-HTTLPR influences the relationship between stress and depression.^30^

The aim of the present study was that to examine in cancer patients the role of 5-HTTLPR in the mental adaptation to the disease, where diagnosis and treatment were considered to be the stressful life events requiring adequate coping by the patients. When the role of 5-HTTLPR in relation to mental adaptation to cancer has been examined by us in a recent previous occasion previously, the baseline HADS and mini-MAC scores measured at recruitment in a different sample of women with early breast cancer were found not to depend on the genetic polymorphism of serotonin transporter.^31^

The evaluation of the patients’ conditions at first assessment (T0) indicates no significant difference in the score of any of the psychometric scales employed when the patients are grouped according to their SERT genotypic variant. An improvement with time, not dependent on 5-HTTLPR, appeared for the avoidance scores of Mini-Mac. The results presented here also indicate that the scores of anxious preoccupation, as identified using Mini-MAC, significantly depended on the genotype of SERT. Moreover, a gene - environment interaction appears for the anxious preoccupation scores, which significantly decreased with the time of follow up, in a way more pronounced in the group of patients carriers of the L/L genetic variant as compared with the carriers of at least one S allele (*r*^2^=0.17 *v.s.* r^2^=0.04, *p*=0.002). The enrolled women were further characterized for the L and S variants of 5-HTTLPR genetic polymorphism, also considering the trial-lelic 5-HTTLPR classification; none of the patients presently investigated had an Lg allele, which is not further considered.

These results support the view that anxious preoccupation plays a significant role in patients with non advanced breast cancer during the early phase of the treatment of the disease. In this connection, several studies showed that an increased anxious preoccupation, as determined with the Mini-MAC scale, was significantly associated with evidence of psychological stress symptoms, and appeared to be the most significant indicator of patients’ difficulties in mental adjustment to cancer.^10^,^32^–^34^ These findings are also in agreement with a feasibility study of psychosocial intervention in women with early breast cancer, which indicated high baseline values of anxious preoccupation HADS scores, which could be reduced by a psycho-educational intervention^35^ based on the approach originally devised by Fawzy and Fawzy for melanoma patients.^36^

The data reported so far indicate that the scores of anxious preoccupation decrease spontaneously with time at follow up, indicating that coping and mental adaptation to the initial diagnosis and to the adjuvant therapy were occurring. The significance of the reduction of avoidance scores appears less clear, since this style of coping has been identified as a risk factor for the poor general mental adjustment and disease progression^37^–^39^, and has been suggested to be adaptive in the short run, but maladaptive for the long-term adjustment.^40^

The decrease with time of anxious preoccupation significantly depended on SERT polymorphism, and was less pronounced in women carrying one or two S alleles; these patients appeared to be those in greater need of intervention. The available literature indicates that psycho-social or psycho-educational interventions^36^,^41^–^44^, or drug treatment^45^–^47^ might be indicated. The data presently reported suggest that the individual molecular-genetic information concerning 5-HTTLPR polymorphism can be usefully considered for identifying the patients at greater risk of anxious preoccupation, and may be considered for the choice of the agent in case of drug treatment on the basis of the pharmacogenetic evidence available. The implications for the choice of the possible treatment of the patients with anxiolytic or antidepressant drugs, which are indicated also for conditions involving an anxious component^48^ as an alternative to psycho-educational intervention, are interesting. The review of the pharmacogenetic of selective serotonin reuptake inhibitor (SSRI) antidepressants indicates that a greater incidence of adverse effects, and presumably a lesser therapeutic response, is likely to occur in carriers of S alleles of 5-HTTLPR variants of serotonin transporter.^45^,^48^ When the drug treatment is the choice of intervention for treating difficulties of mental adaptation to cancer, benzodiazepines or antidepressant agents with a mechanism of action different from that of SSRIs 49,50 should be considered in the perspective of a personalized and more effective treatment. A focussed attention thus might be given to pharmacogenetics, together with consideration to possible pharmacokinetic or pharmacodynamic interactions between the drugs received by the patients^51^, such as those already reported for SSRIs and tamoxifen.^52^,^53^

The further research extended to include a larger cohort of patients, appears to be encouraged by the results reported and is currently being performed by the authors in the perspective of a further investigation of mental adaptation to cancer in relation to the genetic and cultural ethnic milieu of the patients, including the goal of a personalized and more effective intervention.

## Figures and Tables

**TABLE 1.** Socio-demographic and clinical characteristics of the patients

	**Patients N = 48**
**SOCIO-DEMOGRAPHIC CHARACTERISTICS**	
Age (mean±S.E.)	60.2 ± 1.33
Min-max	37 – 73
30–39	1 (2.1%)
40–49	8 (16.7%)
40–59	7 (14.6%)
60–69	27 (56.3%)
70–79	5 (10.4%)

**Employment status**	
Employed	16 (33.3%)
Unemployed	9 (18.8%)
Retired	23 (47.9%)

**Marital status**	
Married	34 (70.8%)
Single	2 (4.2%)
Divorced/Separated	3 (15.8%)
Widowed	8 (16.7%)

**Education completed**	
Primary school	7 (14.6%)
Secondary school	12 (25.0%)
Professional school	9 (18.8%)
High school	20 (41.7%)

**ClINICAl CHARACTERISTICS**	
**Grading**	
1	7 (14.6%)
2	31 (64.6%)
3	10 (20.8%)

**Disease stage**	
-	1 (2.1%)
I	23 (47.9%)
II	20 (41.7%)
III	4 (8.3%)

**Surgery**	
No surgery	2 (4.2%)
Tumorectomy	1 (2.1%)
Quadrantectomy	34 (70.8%)
Mastectomy	11 (22.9)

**Treatment**	
Chemotherapy	16 (33.3%)
Radiation therapy	36 (75%)
Hormonal therapy	34 (70.8%)
Biological therapy	0

**TABLE 2.** 5-HTTLPR allelic variants of the patients

	**Allelic variant**	Functionality*	**N**	**%**
	“short” (S)	Low	3	6.3
**5-HTTlPR**	“short-long” (S/L)	Low	26	54.2
	“long” (L/L)	High	19	39.6

* The functional activity is classified as indicated by Lesch (Lesch et al., 1996)

**TABLE 3.** Subscale scores of MINI-MAC in relation to time and 5-HTTLPR genotype

	**Allelic variants**	**T0 mean±SEM**	**T1 mean±SEM**	**T2 mean±SEM**	**Effect of Time p^a^**	**Effect of genotype p^b^**
**Hopelessness-Helplessness**	S/S-S/L-L/L	11.94±0.58 (N=48)	11.67±0.53 (N=48)	11.06±0.64 (N=35)	.581	.692
	S/S-S/L	11.86±0.71 (N=29)	11.62±0.72 (N=29)	11.63±1.06 (N=19)	.969	
	L/L	12.05±1.01 (N=19)	11.74±0.78 (N=19)	10.38±0.61 (N=16)	.353	
**Fighting spirit**	S/S-S/L-L/L	14.17±0.37 (N=48)	14.25±0.28 (N=48)	14.20±0.47 (N=35)	.986	.455
	S/S-S/L	14.17±0.40 (N=29)	14.28±0.32 (N=29)	14.68±0.57 (N=19)	.698	
	L/L	14.16±0.71 (N=19)	14.21±0.52 (N=19)	13.63±0.78 (N=16)	.803	
**Fatalism**	S/S-S/L-L/L	9.35±0.35 (N=48)	9.04±0.32 (N=48)	8.94±0.31 (N=35)	.663	.520
	S/S-S/L	9.14±0.43 (N=29)	8.97±0.43 (N=29)	8.95±0.44 (N=19)	.942	
	L/L	9.68±0.61 (N=19)	9.16±0.49 (N=19)	8.94±0.44 (N=16)	.595	
**Anxious preoccupation**	S/S-S/L-L/L	15.71±0.70 (N=48)	13.50±0.65 (N=48)	12.51±0.67 (N=35)	.004	.023
	S/S-S/L	16.31±0.96 (N=29)	13.72±0.86 (N=29)	14.21±0.96 (N=19)	.102	
	L/L	14.79±0.99 (N=19)	13.16±1.00 (N=19)	10.50±0.66 (N=16)	.008	
**Avoidance**	S/S-S/L-L/L	10.79±0.47 (N=48)	9.50±0.41 (N=48)	9.43±0.44 (N=35)	.048	.698
	S/S-S/L	11.00±0.65 (N=29)	9.31±0.53 (N=29)	9.68±0.65 (N=19)	.105	
	L/L	10.47±0.65 (N=19)	9.79±0.66 (N=19)	9.13±0.60 (N=16)	.352	

The data reported are the mean±SEM of the psychometric scores at recruitment (T0), after one month (T1) and three months (T2)

The data were analyzed with the analysis of variance (ANOVA), testing the effect of time^a^ and of genetic polymorphism^b^ as independent variables; statistical significance was set at p<0.05 level

**TABLE 4.** Subscale scores of HADS in relation to time and 5-HTTLPR genotype

	**Allelic variants**	**T0 mean±SEM**	**T1 mean±SEM**	**T2 mean±SEM**	**Effect of Time p^a^**	**Effect of genotype p^b^**
**Depression**	S/S-S/L-L/L	4.15±0.46 (N=48)	3.10±0.38 (N=48)	2.97±0.50 (N=35)	.124	.429
	S/S-S/L	4.31±0.60 (N=29)	3.14±0.51 (N=29)	3.32±0.77 (N=19)	.318	
	L/L	3.89±0.75 (N=19)	3.05±0.59 (N=19)	2.56±0.62 (N=16)	.365	
**Anxiety**	S/S-S/L-L/L	4.56±0.59 (N=48)	3.60±0.49 (N=48)	3.37±0.62 (N=35)	.288	.170
	S/S-S/L	5.41±0.83 (N=29)	3.90±0.60 (N=29)	4.21±0.97 (N=19)	.325	
	L/L	3.26±0.69 (N=19)	3.16±0.85 (N=19)	2.38±0.69 (N=16)	.681	

The data reported are the mean±SEM of the psychometric scores at recruitment (T0), after one month (T1) and three months (T2)

The data were analyzed with the analysis of variance (ANOVA), testing the effect of time^a^ and of genetic polymorphism^b^ as independent variables; statistical significance was set at p<0.05 level
